# GPU-accelerated multitiered iterative phasing algorithm for fluctuation X-ray scattering

**DOI:** 10.1107/S1600576721005744

**Published:** 2021-07-30

**Authors:** Pranay Reddy Kommera, Vinay Ramakrishnaiah, Christine Sweeney, Jeffrey Donatelli, Petrus H. Zwart

**Affiliations:** a Applied Computer Science, Los Alamos National Laboratory, Los Alamos, NM 87545, USA; b Department of Electrical and Computer Engineering, University of Wyoming, Laramie, WY 82071, USA; c Center for Advanced Mathematics for Energy Research Applications, Lawrence Berkeley National Laboratory, Berkeley, CA 94720, USA; d Department of Applied Mathematics, Lawrence Berkeley National Laboratory, Berkeley, CA 94720, USA; e Molecular Biophysics and Integrated Bioimaging Division, Lawrence Berkeley National Laboratory, Berkeley, CA 94720, USA

**Keywords:** fluctuation X-ray scattering, multitiered iterative phasing, polar Fourier transform, spherical harmonic transform, GPU acceleration, CUDA programming, HIP programming, NVIDIA GPUs, AMD GPUs

## Abstract

The multitiered iterative phasing (MTIP) algorithm is used to determine the biological structures of macromolecules from fluctuation scattering data. It is an iterative algorithm that reconstructs the electron density of the sample by matching the computed fluctuation X-ray scattering data to the external observations, and by simultaneously enforcing constraints in real and Fourier space. This paper presents the first ever MTIP algorithm acceleration efforts on contemporary graphics processing units (GPUs). The Compute Unified Device Architecture (CUDA) programming model is used to accelerate the MTIP algorithm on NVIDIA GPUs. The computational performance of the CUDA-based MTIP algorithm implementation outperforms the CPU-based version by an order of magnitude. Furthermore, the Heterogeneous-Compute Interface for Portability (HIP) runtime APIs are used to demonstrate portability by accelerating the MTIP algorithm across NVIDIA and AMD GPUs.

## Abstract 2

The paper presents efforts to accelerate the multitiered iterative phasing (MTIP) algorithm on contemporary graphics processing units (GPUs). Application portability is demonstrated by accelerating the MTIP algorithm on NVIDIA and AMD GPUs using a single codebase.

## 1. Introduction   

The study of structures and functionalities of biological macromolecules plays a vital role in understanding their behavior. Fluctuation X-ray scattering (FXS) (Kam *et al.*, 1981 ▸) is an X-ray solution scattering technique used to determine macromolecular structure where multiple identical copies of the sample are exposed to an ultrashort X-ray pulse and the resulting diffraction patterns are collected. By collecting these X-ray snapshots at rates below the rotational diffusion times of the particles, FXS data encode high-resolution structural details unlike standard solution scattering techniques such as small-angle X-ray scattering (SAXS), which mainly captures particle size and bulk shape information, and wide-angle X-ray scattering (WAXS), which can give details of hierarchical structure information or ordering within particles. The advent of X-ray free-electron lasers (XFELs) has made FXS experiments possible (Kurta *et al.*, 2017 ▸; Mendez *et al.*, 2014 ▸, 2016 ▸; Pande *et al.*, 2018*a* ▸,*b* ▸), because they provide sufficiently short and powerful X-ray pulses. In particular, the time taken for XFEL X-rays to interact with and scatter from macromolecules is shorter than the time taken for full rotation of the macromolecule, preventing rotational averaging of the data as seen in SAXS/WAXS (Neutze *et al.*, 2000 ▸).

Early methods for reconstructing macromolecular structure from fluctuation X-ray scattering were based on algebraic phasing (Poon *et al.*, 2013 ▸; Saldin *et al.*, 2011 ▸) and reverse Monte Carlo (Liu *et al.*, 2013 ▸) methods. These methods have limitations such as not being adaptable to determine the molecular structure for general cases, being computationally complex or having difficulties in convergence. More recently, a multitiered iterative phasing (MTIP) algorithm (Donatelli *et al.*, 2015 ▸) has been developed that is able to efficiently reconstruct general biological structures from the FXS data and has desirable convergence properties. In this method, an electron density model is iteratively updated by projection operators to satisfy real-space constraints while matching the generated computed FXS data to the external FXS data (Section 2). In practice, several independent reconstructions are performed with different random starting densities, which are aligned, averaged and interpolated to a Cartesian grid for creating a visual structure of the macromolecules. The MTIP FXS algorithm enabled the first successful 3D reconstructions from experimental FXS data using both single particles per shot (Kurta *et al.*, 2017 ▸) and multiple particles per shot (Pande *et al.*, 2018*a* ▸), and offers higher-resolution reconstruction models than the SAXS/WAXS methods (Podorov *et al.*, 2006 ▸; Svergun *et al.*, 2001 ▸).

With limited XFEL facilities available to researchers, a high cost for operation of these facilities, a significant research demand and limited beam time, it is important for the experiments to be analyzed in near real time to ensure that sufficient data are collected and to increase the throughput of experiments. Determining the 3D structure of the macromolecules as part of the experimental workflow in near real time would allow instantaneous feedback on the quality of the data collected. The feedback obtained can be used to tune the XFEL’s components and operational parameters to collect data of sufficient quantity and significance.

The MTIP algorithm lends itself well to the use of computational resources to accelerate the FXS data analysis to near real time. The iterative use of linear algebraic functions in the MTIP algorithm makes it ideal for achieving speedup on multicore and many-core architectures.

In this paper, we use hardware acceleration to achieve an order of magnitude speedup relative to the central processing unit (CPU)-based MTIP algorithm for a three-dimensional reconstruction of the electron density map of macromolecules from FXS data. We present an accelerated version of the MTIP algorithm implementation on general-purpose graphics processing units (GPGPUs). The MTIP algorithm is ported to NVIDIA graphics processing units (GPUs) using the Compute Unified Device Architecture (CUDA) programming model. We take advantage of the data-level parallelism in the mathematical operations to achieve the speedup.

In addition to the CUDA-based MTIP algorithm, we develop a portable cross-platform MTIP implementation using the C++ Heterogeneous-Compute Interface for Portability (HIP) programming model. The HIP-based MTIP implementation works across the contemporary NVIDIA and Advanced Micro Devices Inc. (AMD) GPUs without making any architecture-specific changes to the code. It is imperative to achieve portability across platforms owing to the advent of different GPU accelerators and programming models from different vendors. Therefore, we present a comparison of the performance of the MTIP algorithm using CUDA and HIP on NVIDIA GPUs. In addition, we develop a Python-based numerical validation tool to numerically estimate the overall quality of the reconstruction.

## 2. MTIP algorithm   

### 2.1. Overview   

The MTIP algorithm is an iterative process of repeatedly updating an electron density map of a macromolecule to make it consistent with real-space constraints, and enforcing the constraint that the computed FXS data derived from the density map match the external FXS data. The relation (Donatelli *et al.*, 2015 ▸) between the angular correlation function obtained from the diffraction images and the harmonic coefficients of the intensities is used to obtain the external FXS data (supplementary Section S1).

The algorithm is devised using a spherical harmonic basis for three-dimensional functions as described by Donatelli *et al.* (2015 ▸). A 3D spherical polar grid as shown in Fig. 2 (see Section 5) is used, where the radial components in real space *r* and Fourier space *q*, as well the azimuthal angles ϕ, are equispaced. The inclination angles θ lie on the arccosines of Gauss–Legendre quadrature nodes.

The relation between the cross-correlation data *B*_*l*_(*q*, *q*′) and the intensity spherical harmonic coefficients is used to derive the computed FXS data:
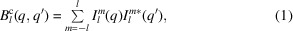
where 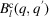 are the computed FXS data, 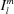 are the intensity spherical harmonic coefficients of order *l* and degree *m*, and * represents the conjugate of the quantity.

The intensity spherical harmonic coefficients and the computed FXS data can be derived from the electron density (supplementary Fig. S2). This process is referred to as the ‘forward direction’ in the rest of the paper. In the forward direction, the electron density ρ(*r*, θ, ϕ) in real space is transformed into structure factors 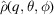 using the polar Fourier transform (Section 2.2). The intensity *I*(*q*, θ, ϕ) is the square magnitude of the structure factors. The intensity spherical harmonic coefficients 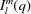 are computed from the intensity function using the spherical harmonic transform (Section 2.2). The obtained intensity spherical harmonic coefficients are used to derive the computed FXS data using equation (1).

Similarly to obtaining FXS data from the electron density, the electron density in real space is updated by applying a series of projection operators starting from the FXS data (supplementary Fig. S3), which is referred to as the ‘inverse direction’ in the rest of the paper. The inverse direction is used to compute an updated electron density map from the FXS data. In the inverse direction, the intensity spherical harmonic coefficients 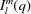 are computed from the FXS data as part of the correlation projections (Section 2.2). The intensity function *I*(*q*, θ, ϕ) is derived from the intensity spherical harmonic coefficients using the inverse spherical harmonic transform (Section 2.2). The structure factors are obtained from the intensity function using a magnitude projection operator, by preserving the phase information. The electron density is obtained from the structure factors using the inverse polar Fourier transform (Section 2.2).

The forward direction, correlation projection, inverse direction and real-space projection constitute one iteration of the MTIP algorithm. The final electron density obtained from an iteration is used as the initial electron density for the next iteration.

### 2.2. MTIP pipeline   

**Figure 1:**
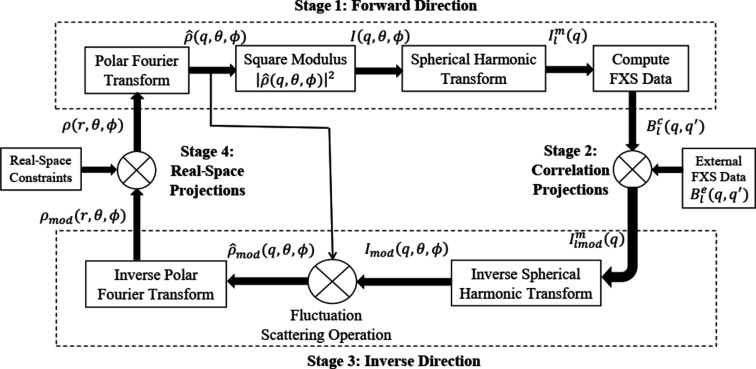
Flowchart of the multitiered iterative phasing algorithm.

The MTIP algorithm can be categorized into four stages as shown in Fig. 1 ▸: Stage 1 – the forward direction of obtaining computed FXS data 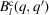 from the electron densities ρ(*r*, θ, ϕ), where the superscript c indicates that this is the computed quantity; Stage 2 – matching the external FXS data 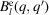 to the computed FXS data by correlation projectors, resulting in modified intensity spherical harmonic coefficients 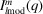, where mod indicates ‘modified quantity’ and e ‘external quantity’; Stage 3 – the inverse direction of obtaining an updated electron density ρ_mod_(*r*, θ, ϕ) from the intensity spherical harmonic coefficients; Stage 4 – imposing the real-space constraints by real-space projectors. The real-space constraints are imposed in real space and the FXS data are matched in the Fourier space.

The electron density, intensity function and spherical harmonic coefficient quantities of the polar nodes can be aligned in the computing memory as vectors/arrays. All the respective transforms can be applied on these arrays and vectors efficiently if the quantities are arranged in contiguous memory. The forward and inverse directions involve various mathematical operations such as the polar Fourier transform, square modulus, spherical harmonic transform, inverse spherical harmonic transform and inverse polar Fourier transform.

The spherical harmonic transforms can be efficiently computed from a given function by a combination of the Fourier transform and the associated Legendre transform (Schaeffer, 2013 ▸) [supplementary equations (S4)–(S6) and Fig. S4]. The Fourier transform is computed using the fast Fourier transform operation, and the associated Legendre transform can be computed using matrix–matrix multiplications on the quantities stored as vectors/arrays.

Similarly, the inverse spherical harmonic coefficients can be efficiently computed from the given function by a combination of the inverse Fourier transform and the inverse associated Legendre transform (Schaeffer, 2013 ▸) [supplementary equations (S7)–(S9) and Fig. S5]. The inverse Fourier transform is computed using the inverse fast Fourier transform, and the inverse associated Legendre transform can be computed using matrix–matrix multiplications on the quantities stored as vectors/arrays. The spherical harmonic transform and its inverse are computed independently for each radius, *r* or *q*.

In addition, the relation between the spherical Hankel transform (Donatelli *et al.*, 2015 ▸) and the spherical harmonic coefficients of a function in real and Fourier space are exploited to compute the polar Fourier transform and inverse polar Fourier transform as shown in supplementary Figs. S6 and S7.

Apart from the forward and the inverse directions, the projectors in real space and Fourier space are used to impose the constraints on the quantities. Correlation projectors (Donatelli *et al.*, 2015 ▸) are used to match the computed FXS data and the external FXS data (supplementary Section S4; Gower & Dijksterhuis, 2004 ▸). In addition, various physical constraints (Donatelli *et al.*, 2015 ▸), such as a finite support, symmetry, lower and upper bounds, and nonnegativity, can be imposed on the electron density using the real-space projectors.

### 2.3. MTIP iterations   

The operations described in Fig. 1 ▸ can be combined in a number of different ways. Here we apply all these operations iteratively using generalizations of both the error-reducing (ER) (Gerchberg, 1972 ▸) and the hybrid input–output (HIO) (Fienup, 1978 ▸) methods, as described by Donatelli *et al.* (2015 ▸). In particular, HIO is a global optimization technique that uses negative feedback to prevent stagnation into local minima. In contrast, ER is a local minimizer used to refine a solution and consists of simply applying the operations in Fig. 1 ▸ in sequential order without the use of negative feedback.

Both the ER and HIO methods are used for a set number of iterations one after the other to improve the convergence of the electron density. These methods are combined with shrinkwrap (Marchesini *et al.*, 2003 ▸) to update estimates of the density support region which is enforced in the HIO and ER steps. The pseudocode described below represents how HIO, ER and shrinkwrap are alternated within the MTIP algorithm implementation and is called the iterative stage in the rest of the paper.

Alogrithm 1.
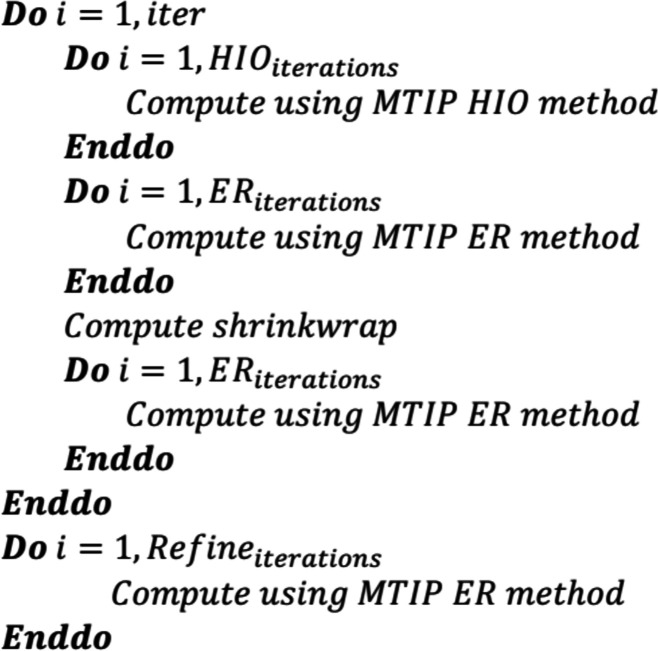


## 3. Literature review   

Modern GPGPUs targeting high-performance computations are widely used in various scientific applications. The lightweight properties of the processing cores, which are large in number on GPGPUs, make them ideal for large-scale arithmetic operations. In contrast, CPUs with their complex logical circuitry are suitable for serial and conditional instructions. In many scientific applications, GPUs are used as co-processors for compute-intensive and time-consuming arithmetic operations.

In the past decade, GPUs have been successfully used in various structure analysis computations. Single-particle electron microscopy (Schmeisser *et al.*, 2009 ▸) was one of the first areas to introduce the use of GPU computing technologies for determination of the structure of macromolecules. The parallelism offered by GPUs is exploited in computation of diffuse scattering patterns (Gutmann, 2010 ▸), Debye function analysis (Gelisio *et al.*, 2010 ▸; Sestu *et al.*, 2014 ▸), computation of scattering maps (Favre-Nicolin *et al.*, 2011 ▸) and many other applications.

Various diffraction data analysis applications have recently shown a significant performance boost when using GPUs. The *SHARP* package (Marchesini *et al.*, 2016 ▸) and multimode ptychography (Dong *et al.*, 2018 ▸) use GPUs to achieve high-throughput ptychographic (Hegerl & Hoppe, 1970 ▸) reconstructions. A GPU version of the *TREOR* algorithm (Šimeček *et al.*, 2015 ▸) was developed for indexing powder diffraction data. Other applications such as atom-based polychromatic diffraction simulation (E *et al.*, 2018 ▸) have effectively utilized GPUs to accelerate computations.

In addition to the use of GPUs to achieve higher computational throughput in the algorithms, they have been employed in data classification and processing techniques such as classification algorithms for XFEL data (Shi *et al.*, 2019 ▸), data reduction (Narayanan *et al.*, 2018 ▸) and identifying useful diffraction patterns for further study (Cichocka *et al.*, 2018 ▸). Various libraries and toolbox packages like the *Astra Tomography Toolbox* (Palenstijn *et al.*, 2011 ▸), *pyFAI* (Ashiotis *et al.*, 2015 ▸), *crYOLO* (Wagner *et al.*, 2020 ▸) and *SIR2011* software algorithms (Shalaby & Oliveira, 2013 ▸) utilize GPUs for efficient parallel calculations.

## 4. GPU programming models   

Various parallel programming models are available for programming GPGPUs. We chose CUDA (Nvidia, 2010 ▸) and HIP (AMD, 2016 ▸) for our implementations. CUDA is an application programming interface (API) model used to program NVIDIA GPUs. The CUDA programming model includes a large set of accelerated libraries for linear algebra, signal and image processing, and deep learning frameworks, which are highly optimized and ready to use.

HIP is a runtime API and kernel language used to create a portable application that can execute on both the NVIDIA and AMD GPUs. HIP also has support for various libraries. HIP APIs implicitly invoke the CUDA library APIs when executing on NVIDIA GPUs and invoke APIs from the ROCm stack (ROCm, 2016 ▸) when executing on AMD GPUs. The CUDA and HIP APIs are syntactically similar with different prefixes (supplementary Table S1).

## 5. MTIP application characteristics and CPU-based profiling   

**Figure 2:**
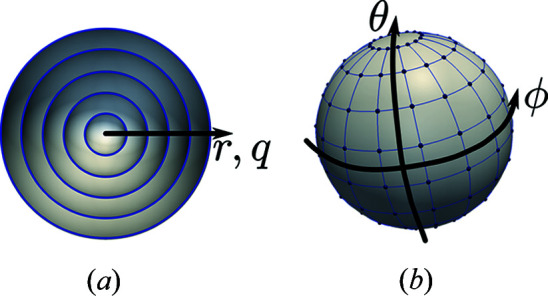
Spherical polar grid, where *r* and *q* are radii in real and Fourier space, and θ and ϕ are inclination and azimuthal angles.

In the MTIP algorithm, the functions are discretized into polar nodes/points, which are used to approximate the integral. The 3D spherical-polar grids used in MTIP consist of *N* spherical shells as shown in Fig. 2 ▸(*a*) at equispaced radii *r*_1_,…, *r*_*N*_ in real space and *q*_1_,…, *q*_*N*_ in Fourier space. The *n*th spherical shell, as shown in Fig. 2 ▸(*b*), contains *L*_*n*_ inclination angles 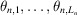, which are given by the arccosines of the *L*_*n*_th-order Gauss–Legendre quadrature nodes, and *M*_*n*_ equispaced azimuthal angles 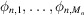. In real space, the values of *L*_*n*_ and *M*_*n*_ differ for each spherical shell and are chosen to balance between maximizing the accuracy of the spherical harmonic transforms and minimizing the number of degrees of freedom used to represent the solution (*i.e.* by preventing the grid point density from blowing up at the center). In the current implementation *L*_*n*_ = π*n* + 1 and *M*_*n*_ = 2*L*_*n*_ − 1. However, in Fourier space, the values of *L*_*n*_ and *M*_*n*_ are instead set to the same constants *L* and *M* for each spherical shell in order to fully maximize the accuracy of the spherical harmonic transforms, since the degrees of freedom have already been limited by the real-space representation. In the current implementation we use *L* = π(*N* − 1) + 15 and *M* = 2*L* − 1.

All the quantities on the spherical polar grid are stored contiguously in computing memory before the respective operations. The contiguous arrangement of the quantities in memory results in effective coalesced memory access. As a result, the quantities are reordered before every mathematical operation such that they are contiguous with respect to parameters like inclination angle and azimuthal angle. The associated Legendre transforms (ALT), Hankel transforms (HT), and their inverses IALT and IHT involve the use of matrix–matrix multiplications. The Fourier transforms involved in polar Fourier transforms, the spherical harmonic transforms and their inverses are computed using the fast Fourier transform (FFT) and an inverse fast Fourier transform (IFFT).

Each iteration of the MTIP algorithm involves computing a set of mathematical operations in a sequential order as shown in Fig. 1 ▸, to obtain an updated electron density. The outputs from each mathematical operation are reordered to be contiguous with respect to one of the parameters. This reordering results in contiguous memory access and efficient computation of fast Fourier transforms and matrix–matrix multiplications. The MTIP algorithm also involves other computations (Donatelli *et al.*, 2015 ▸) including singular value decompositions, eigen-decompositions, the shrinkwrap method and other mathematical operations while enforcing the projections using mathematical operators.

**Figure 3:**
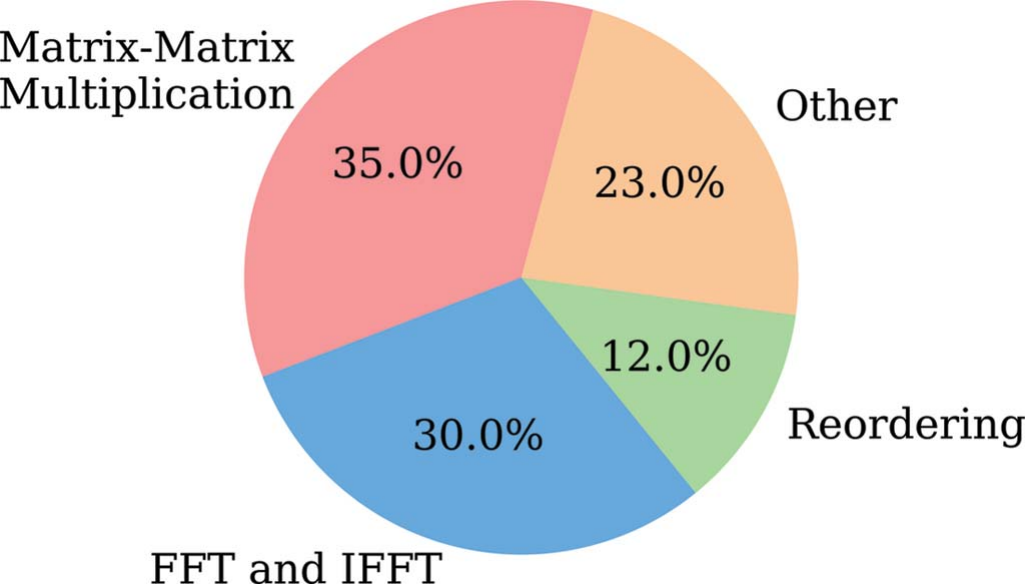
Pie chart of the timing profile for different mathematical sections of the iterative stage in the CPU-based MTIP algorithm implementation.

Fig. 3 ▸ shows a pie chart representation of the timing profile for the CPU-based implementation of the iterative stage in the MTIP algorithm, derived using Intel *VTune Amplifier* (Intel, 2020 ▸). The iteration parameters as represented in Algorithm 1 used for the iterative stage of the MTIP algorithm implementation in this paper are iter = 15; HIO_iterations_ = 60; ER_iterations_ = 40; Refine_iterations_ = 200. The mentioned iteration parameter configuration is used as it has provided good convergence. From Fig. 3 ▸, it is evident that the FFT, IFFT and matrix–matrix multiplications are the major bottlenecks in the implementation.

Owing to the varied values of the angles in both the real space and the Fourier space, the matrix–matrix multiplications and the fast Fourier transforms consist of varied array/vector sizes for each value of *n*. The MTIP algorithm is implemented using double-precision floating-point arithmetic to eliminate small numerical roundoff errors on the order of 10^−10^, which would grow after several iterations and eventually cause the iterations to become unstable. The algorithm executes independently for various different initial electron densities by distributing the code to multiple message passing interface (MPI) (Gropp *et al.*, 1999 ▸) ranks and randomly initializing the electron density. The obtained reconstructions from each of the MPI ranks are averaged and interpolated to a Cartesian grid for visualization. A higher number of reconstructions results in improved model accuracy.

## 6. Methods   

In this section, we describe the details of the GPU acceleration of MTIP, the hardware and libraries used for our performance study, and the validation tool that we developed. We use the CUDA programming model, because it is a low-level language targeting NVIDIA GPUs effectively, and it can provide a good baseline for the best GPU performance possible. We show portability to AMD GPUs via HIP owing to its similarity to CUDA syntax and for its ease of porting.

The performance of the GPU-accelerated MTIP algorithm implementation is demonstrated by reconstructing the *Paramecium bursaria Chlorella* virus (PBCV-1) (Van Etten *et al.*, 1983 ▸) from experimental data (Pande *et al.*, 2018*a* ▸,*b* ▸), and the *Methano­coccus marapaludis* archeal chaperonin (MMAC) (Zhang *et al.*, 2010 ▸) protein from simulated data.

### 6.1. GPU acceleration of MTIP   

#### 6.1.1. Initialization stage   

The MTIP code implementation has an initialization stage followed by an iterative stage. The initialization stage involves initializing parameters for polar Fourier transforms, spherical harmonic transforms and their inverses by creating plans for fast Fourier transforms and the execution environment for matrix–matrix multiplications. The polar Fourier transforms, spherical harmonic transforms and their inverses involve the use of various special function evaluations, which are used in the transforms. These functions are evaluated once in the initialization stage and used in every iteration of the MTIP algorithm. To compute fast Fourier transforms, all the libraries targeting CPUs or GPUs require plan creation. The plan is a data structure that stores all the information required to compute the transforms. Since the plans do not change between iterations of the MTIP algorithm, they are created once in the initialization stage. Similarly, the dimensions of various matrices generated in the MTIP algorithm are constant. Abstractions for various matrix–matrix operations are created in the initialization stage by using user-defined datatypes (classes) in C++.

#### 6.1.2. Fast Fourier transforms   

Fourier transforms and their inverses in the MTIP algorithm involve real-to-complex, complex-to-real and complex-to-complex transforms. The complex-to-complex transforms in the cuFFT library (NVIDIA, 2013 ▸) handle the entire transform effectively using optimized kernels. However, the library involves the use of different algorithms for the real-to-complex and complex-to-real transforms depending on the input size.

In addition, the pointers to the input and output of the transforms for the real-to-complex and complex-to-real transforms in cuFFT are required to be aligned to the complex data type (NVIDIA, 2013 ▸) and otherwise will throw an invalid error. Therefore, the starting address of the input real type and output real type should be in the even address space, which is not the case in the MTIP algorithm.

**Figure 4:**
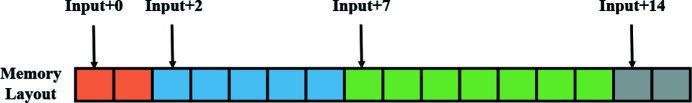
Memory layout of different vectors.

For example, let us consider different sub-vectors (in different colors) arranged linearly in memory and accessed as a single vector as shown in Fig. 4 ▸. The cuFFT library can readily access Input+0 and Input+2 memory locations as they are aligned to the complex data type. But the Input+7 memory location cannot be accessed by the library and will throw an error as it is not aligned to the complex data type.

**Figure 5:**
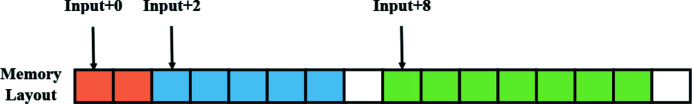
Padded memory layout for different sub-vectors.

Three possible workarounds have been explored. In the first technique, temporary vectors are initialized with the size of each sub-vector, and the sub-vectors are copied into them. Each temporary vector is passed as an argument to the cuFFT API calls. This results in additional initialization and data transfers. The second technique consists of padding the vector such that each sub-vector is in the even address space as shown in Fig. 5 ▸. The padded memory layout will result in a change of the input vector size. The third technique consists of converting all the real data types into complex data types by adding a zero-valued imaginary component. The output of the real-to-complex transforms will then be equal to the first half of the output vector from the complex-to-complex transform. By converting the real data type to a complex data type, the memory usage in the FFT-related computations was doubled.

The third technique resulted in around 25% better performance compared with the second technique. And as the memory increase by converting the real data type to the complex data type is significantly lower than the entire application’s memory requirement, we implemented the third technique. As a result, we reorganized all the real-to-complex and complex-to-real transforms as complex-to-complex transforms during the reordering step.

#### 6.1.3. Matrix–matrix multiplication   

We implemented the matrix–matrix multiplications in the GPU-accelerated MTIP algorithm using the cuBLAS library (Nvidia, 2008 ▸). Each of the matrix–matrix multiplications is independent across order *l* and *m*. These independent matrix–matrix multiplications can be executed asynchronously. Therefore, we use CUDA streams to execute each matrix–matrix multiplication asynchronously.

#### 6.1.4. Reordering   

Data dependencies across different mathematical operations in each iteration and the iterations themselves restrict the GPU-based computations to targeting each mathematical operation and iteration individually one after the other. In each of the mathematical operations, the inputs and outputs are reordered such that they are contiguous in terms of various parameters, including radial component, expansion order, polar angle, azimuthal angle, real space and Fourier space. This contiguous reordering at each step within an iteration results in effective memory access patterns for the Fourier transforms and matrix–matrix multiplications. Unlike in fast Fourier transforms and matrix–matrix multiplications where respective cuBLAS and cuFFT library API calls are invoked, we have developed all the reordering modules using manually written CUDA kernels.

Note that MTIP uses a small (80–95) number of radial nodes *N* and relatively small (size of *M*_*n*_) array sizes in matrix–matrix multiplications and vector sizes in the FFT and IFFT. Profiling of the mathematical operations by the NVIDIA *Profiler* (NVIDIA, 2020 ▸) shows that the achieved occupancy of the GPU is lower than 15%. This lower achieved occupancy means that the GPU is not being used at its maximum computational capability and is due to fewer computations being performed on the GPU because of the smaller array/vector sizes. Despite the fact that fewer computations are performed on the GPU when compared with its capacity, each mathematical operation is computed on the GPU independently in a sequence because of their dependency across the operations in the algorithm.

### 6.2. Hardware and libraries   

We ran the accelerated MTIP algorithm using 96 ranks on the Summit supercomputer (https://www.olcf.ornl.gov/summit/). Summit nodes have IBM Power9 CPUs and six NVIDIA Tesla V100 GPUs. Each Tesla V100 GPU has a 16 GB memory limit. The accelerated portions of the algorithm are executed on the Tesla V100 GPUs and the non-accelerated portions of the algorithm on the Power9 CPUs.

We demonstrate the cross-platform implementation by executing the accelerated MTIP algorithm with one rank using HIP on the NVIDIA Tesla V100 and AMD Radeon Instinct MI50 GPUs. The accelerated portions of the algorithm are executed on the AMD MI50 GPUs, and AMD EPYC CPUs are used for running the non-acceleration portions of the algorithm when employing HIP on the AMD GPU.

As the MTIP algorithm consists of a significant number of matrix–matrix multiplications, FFTs and IFFTs, the use of highly optimized libraries targeting specific architectures would be beneficial for the performance. Therefore, we use cuBLAS (Nvidia, 2008 ▸) and cuFFT (NVIDIA, 2013 ▸) in our CUDA implementation and hipBLAS and hipFFT (https://github.com/ROCmSoftwarePlatform) in our HIP implementation. The HIP runtime and library APIs implicitly invoke their CUDA counterparts on NVIDIA GPUs, resulting in a portable application. Other mathematical computations like reordering, shrinkwrap and projection operators are implemented by explicitly developing CUDA/HIP kernels on the NVIDIA/AMD GPUs.

### 6.3. Validation   

The GPU-accelerated MTIP algorithm uses multiple MPI ranks on multiple nodes with each rank executing the algorithm independently, starting with different initial electron densities. The final electron densities (reconstructions) obtained from each rank are then aligned, averaged and interpolated to a Cartesian grid for visualization. In addition to the visualization, the phase retrieval transfer function (PRTF) (Marchesini *et al.*, 2005 ▸) values are computed to validate the resolution of the reconstruction with respect to an initial reconstruction obtained using sequential computations.

The PRTF is a one-dimensional curve, which is a function of resolution and can be used to estimate the overall quality of the reconstruction. The estimated resolution of the reconstruction is provided as the first PRTF value that is below a threshold of 1/*e*.

We numerically validate the reconstruction by checking if all the PRTF values above 0.25 have less than ±5% variation compared with the initial reconstruction. We developed a Python-based numerical validation tool to validate the final reconstructions obtained. The validation tool compares the PRTF values obtained from the GPU-accelerated MTIP algorithm with the PRTF values of the CPU-based MTIP algorithm and visually represents the variations.

## 7. Results   

**Figure 6:**
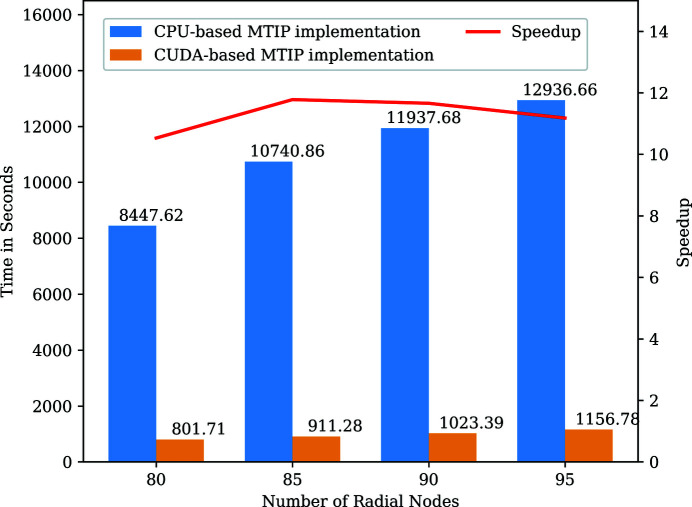
Timing comparison of iterative stage on Power9 CPUs and NVIDIA Tesla V100 GPUs.

We ran the CUDA-based and the CPU-based MTIP algorithms for different numbers of radial nodes, and the execution time taken by 96 MPI ranks for computing the iterative stage for the PBCV-1 virus is shown in Fig. 6 ▸. The time displayed is for the iterative stage as shown in Algorithm 1 with the iteration parameters given in Section 5.

The CPU-based code was executed on the Power9 architecture. Various optimization techniques were employed to improve the performance of the CPU-based MTIP algorithm. Among the compiler-based optimizations, the XL compiler was used with the level-3 compiler optimization flag to perform high-order transformations on loops along with loop unrolling, which improved the performance compared with the use of the PGI compiler. In addition, various optimizations were employed in both the initialization and iterative stages of the algorithm. In the initialization stage, the preliminary computations of the special function evaluations, which involve trigonometric functions and other mathematical computations, require a significant amount of time and are identical across the MPI ranks. As a result, these preliminary computations were evaluated and written into a file to be read during the initialization stage. Reading the preliminary input from a file reduces the time of the initialization stage by 45%. In the iterative stage, various optimization techniques such as minimizing the recurring computations by storing them in scalar variables for further use and contiguous memory storage for effective memory access were implemented to improve performance. These techniques along with the XL compiler-based optimizations improved the performance of the iterative stage by around 15%. The use of Intel Broadwell CPUs with Intel compilers resulted in 18% improvement in performance of the CPU-based MTIP implementation compared with the Power9 CPUs. To maintain homogeneity, results from Power9 CPUs are compared for the CPU-based and CUDA-based implementations.

**Figure 7:**
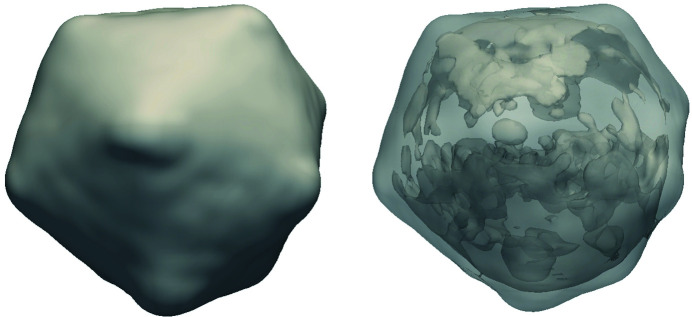
Reconstruction of PBCV-1 virus using the MTIP CUDA implementation.

**Figure 9:**
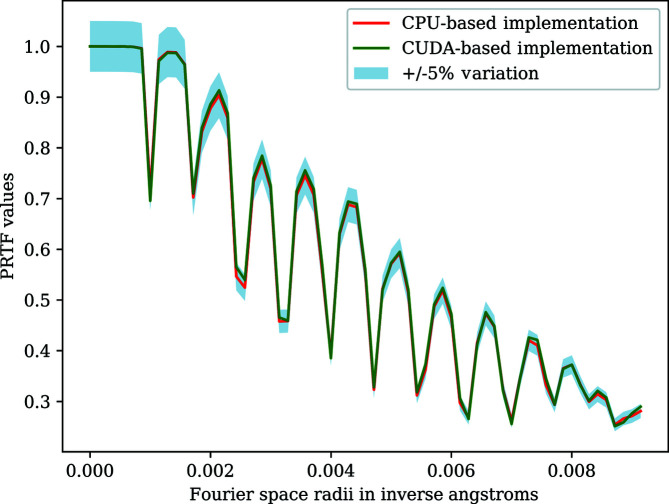
MTIP numerical validation using a Python-based validation tool, showing that the PRTF values of the CUDA-based implementation are within 5% of those of the CPU-based implementation.

The CUDA-based MTIP algorithm was executed using 96 MPI ranks distributed on 16 nodes of the Summit supercomputer (which has six Tesla V100 GPUs per node) with six MPI ranks per node and one MPI rank per GPU. The initialization stage uses 96 MPI ranks and was executed on the Power9 CPU cores, and the iterative stage was offloaded to the GPUs. The CUDA implementation achieved a speedup of approximately one order of magnitude for different numbers of radial nodes. Figs. 7 ▸ and 8 ▸ show the final reconstructions of virus/protein obtained using the CUDA-based implementation. We compared the numerical validation of the CUDA-based implementation with the CPU-based implementation using the Python validation tool, and the PRTF values of the GPU implementation are well within the ±5% variation, as shown in Fig. 9 ▸.

Owing to memory limitations on GPUs, the CUDA-based MTIP algorithm cannot run more than 95 radial nodes. Even though it is possible to run the CPU-based MTIP algorithm with more than 95 radial nodes, we did not see any further improvement in the quality of reconstruction when doing so. However, it increased the computational load significantly. Therefore, we only show results up to 95 radial nodes.

We initially ported the iterative stage completely to GPUs, but the correlation projection on GPUs had a negative impact on the performance. This is due to synchronous execution of the singular value decomposition on smaller-dimensional matrices. As a result, we modified the code to execute the correlation projection on the CPU by copying data to and from the CPU, before and after the correlation projection.

To achieve performance portability across different GPU architectures (NVIDIA and AMD), we ported the CUDA-based code to use the HIP programming interface. The HIP interface has support for BLAS (hipBLAS) and FFT (hipFFT) libraries but they are not as performant (at the time of this acceleration effort) as their CUDA counterparts. As a result, the HIP-based MTIP code on AMD GPUs takes considerably more time than the CUDA-based MTIP code on NVIDIA GPUs. The execution time of the HIP-based MTIP code on AMD GPUs is around six times slower than that of the CUDA-based MTIP code on NVIDIA GPUs.

**Figure 10:**
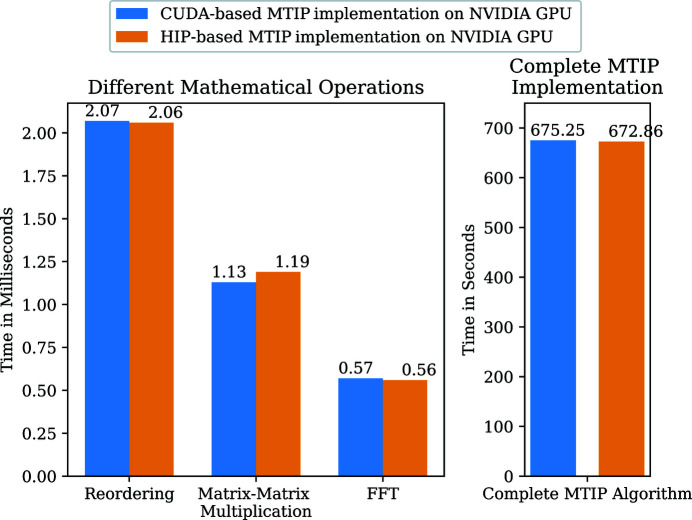
Timing comparison of CUDA and HIP programming models on NVIDIA GPUs.

The HIP-based MTIP code on the NVIDIA GPUs has similar performance to the CUDA-based MTIP code on the NVIDIA GPUs. Fig. 10 ▸ shows that both the HIP and CUDA implementations have similar performance on NVIDIA GPUs. Although, at present, the performance of HIP libraries on AMD GPUs is not comparable to that of CUDA libraries on NVIDIA GPUs, developing the code in HIP will readily provide us with a portable code without any performance penalty on the NVIDIA GPUs. Further improvements to the HIP libraries in the future will implicitly improve the performance of GPU MTIP code on the AMD GPUs.

## 8. Discussion   

In our experiments, the number of radial nodes was varied (supplementary Section S5) between 80 and 95. The parameters including inclination angles and azimuthal angles in real/Fourier space depend on the number of radial nodes. As a result, a change in the number of radial nodes will implicitly change all the parameters in the MTIP algorithm.

**Figure 11:**
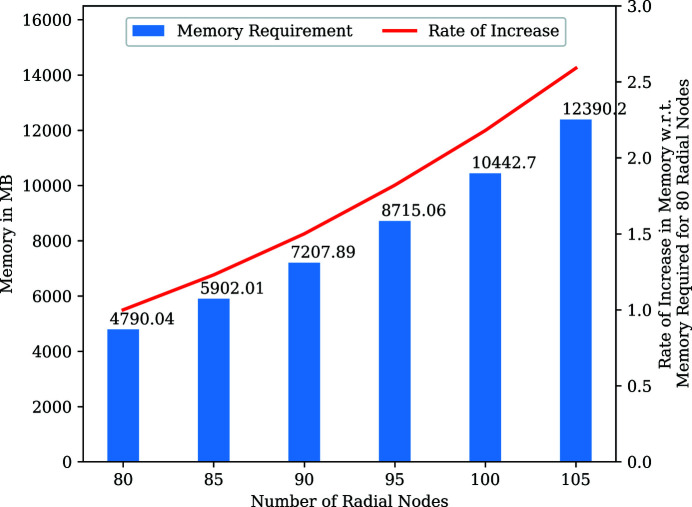
Memory requirement in the MTIP algorithm for varying number of radial nodes, and rate of increase in memory with respect to 80 radial nodes.

With the increase in number of nodes, there is an increase in the vector sizes of variables involved in the various transforms and matrix–matrix multiplications. In addition, the total number of FFTs, IFFTs and matrix–matrix multiplication operations also increases with increasing number of radial nodes. Fig. 11 ▸ provides information about the approximate memory required by the MTIP algorithm for various numbers of radial nodes and the rate at which the memory requirement increases. The rate of increase in memory requirement was compared with the memory requirement of the MTIP algorithm for 80 radial nodes. Fig. 11 ▸ shows that, as the number of radial nodes increases, the memory required for the MTIP algorithm increases significantly. The plot numerically indicates that a 30% increase in the number of nodes increases the memory requirement by more than two times.

From a computational perspective, the occupancy of the GPUs is less than 30–40%, because the algorithm is restricted to computing each mathematical operation in a serial order. Increasing the number of radial nodes will increase the vector sizes and the number of operations, resulting in improved occupancy of the GPUs. The increase in occupancy of the GPUs would generally improve the computational throughput, but in the MTIP algorithm the increase in vector sizes is lower. This is because the number of radial nodes is increased in small proportions, resulting in a lower increase of occupancy in the GPUs. To achieve higher occupancy, the number of radial nodes must be increased significantly, which leads to a memory limitation on the GPUs. On the other hand, reducing the number of radial nodes to below 80 would further decrease the occupancy of the GPUs. The MTIP algorithm can be categorized as a memory-bound problem, where higher memory access is required for fewer computations. As a result, the number of radial nodes must be chosen such that better output reconstructions can be obtained from a lower number of radial nodes, which would involve possible higher occupancy on the GPUs.

Fig. 6 ▸ shows that the speedup does not change significantly with an increase in the number of radial nodes. The speedup of the mathematical operations like transforms and matrix–matrix multiplications individually ranges from 5× to around 30×. With the increase in the number of radial nodes, the speedup of these individual operations would increase further but not significantly owing to the lower increase in the number of radial nodes. As these operations are cumulated serially as part of the MTIP iteration, and a significantly larger number of iterations are executed, the overall speedup improvement is not significant. From the available timing results, it can be observed that with the increase in the number of radial nodes the time increases linearly in both the CPU-based and the GPU-based implementations. But the speedup does not change significantly, indicating that the rate of change of time in CPU-based implementation for a varied number of radial nodes is proportional to the rate of change of time in GPU-based implementation for a varied number of radial nodes.

## 9. Conclusion   

In this paper, we presented GPU acceleration efforts for the multitiered iterative phasing algorithm using the CUDA programming model. We detailed the limitations of the real-to-complex and complex-to-real fast Fourier transforms and their inverses in the CUDA libraries particular to MTIP data and proposed different workarounds. The CUDA-based MTIP fluctuation X-ray scattering analysis program outperforms the CPU-based version by an order of magnitude. In addition to the CUDA implementation, we developed a portable application using the HIP programming interface. Although the performance on AMD GPUs is restricted by the HIP libraries, we demonstrated a pathway to use the HIP interface to develop a cross-platform application for NVIDIA and AMD GPUs.

## Supplementary Material

Additional supporting information for the paper. DOI: 10.1107/S1600576721005744/cw5031sup1.pdf

Zipped copy of the code. DOI: 10.1107/S1600576721005744/cw5031sup2.zip

## Figures and Tables

**Figure 8:**
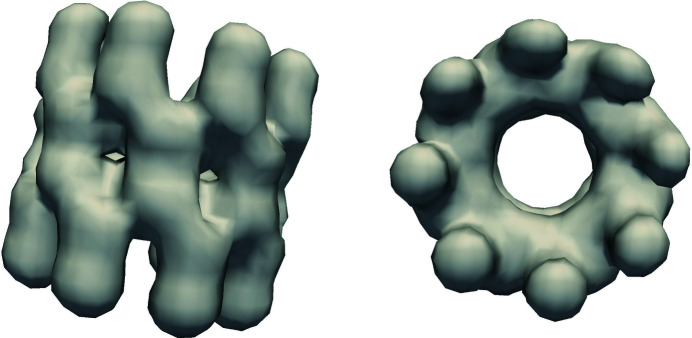
Reconstruction of MMAC protein using the MTIP CUDA implementation.
