# Supplementary material for: GPU-accelerated multitiered iterative phasing algorithm for fluctuation X-ray scattering
Source: J Appl Crystallogr. 2021 Jul 30;54(Pt 4):1179–88. doi: 10.1107/S1600576721005744 (PMC8366419; doi:10.1107/S1600576721005744)
Supplement: Supplementary file 1 [file j-54-01179-sup1.pdf]

JOURNAL OF APPLIED CRYSTALLOGRAPHY

Volume 54 (2021)

Supporting information for article:

GPU-accelerated multitiered iterative phasing (MTIP) algorithm for fluctuation X-ray scattering

Pranay Reddy Kommera, Vinay Ramakrishnaiah, Christine Sweeney, Jeffrey Donatelli and Petrus H. Zwart

Supporting Information

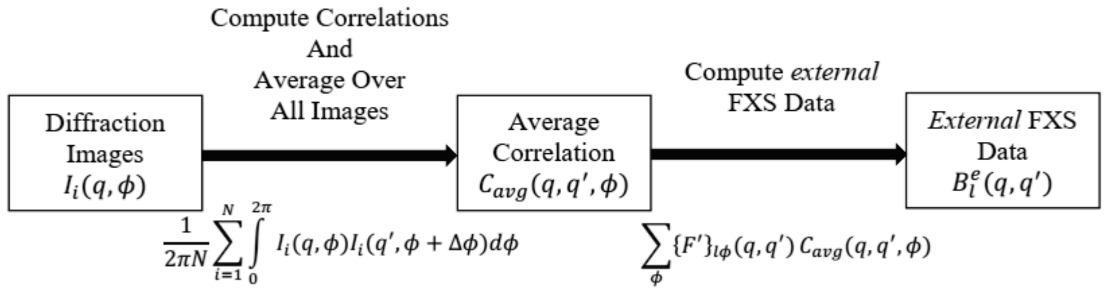

Figure S1 Flowchart of the process of computing *external* FXS data, provided the diffraction images.

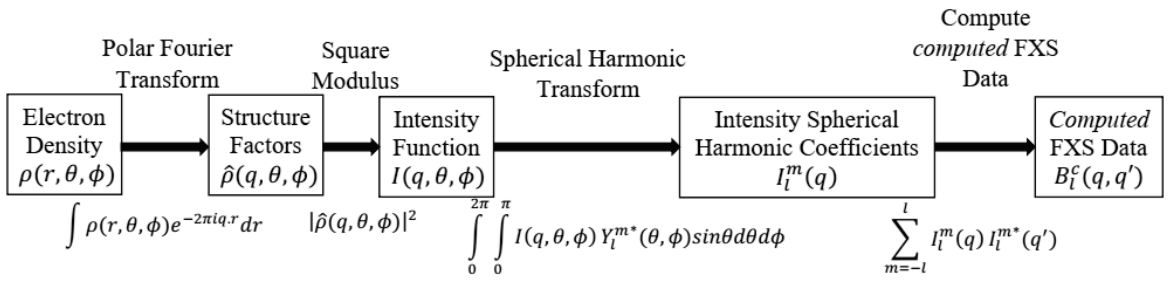

Figure S2 Forward Direction: Flowchart of the process of computing fluctuation scattering data, provided the electron density.

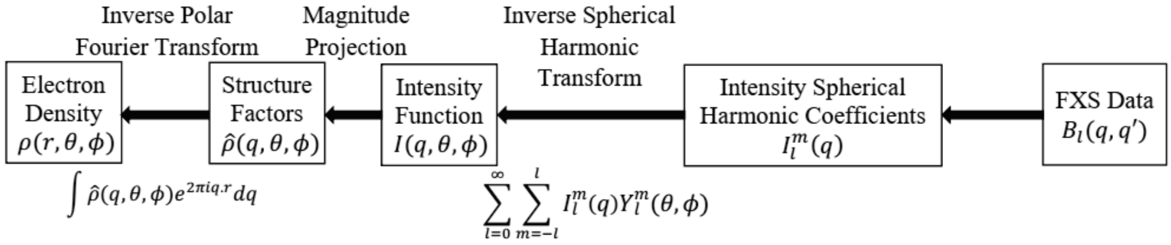

Figure S3 Inverse Direction: Flowchart of the process of updating the electron density, provided the fluctuation scattering data.

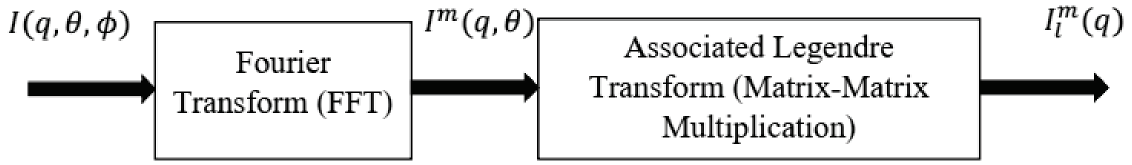

Figure S4 Flowchart of Spherical Harmonic Transform.

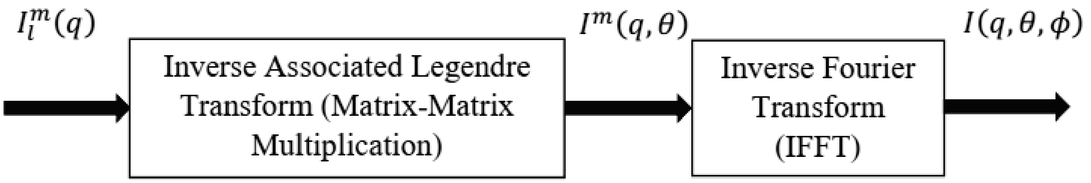

Figure S5 Flowchart of Inverse Spherical Harmonic Transform.

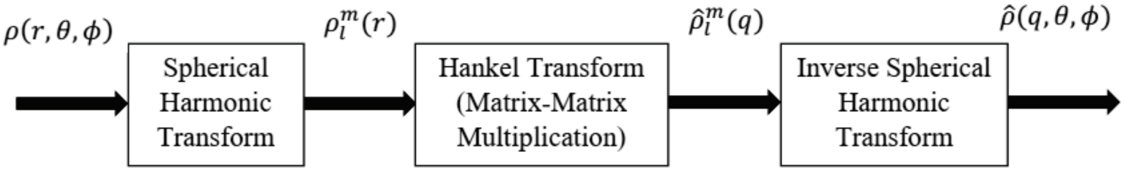

Figure S6 Flowchart of Polar Fourier Transform.

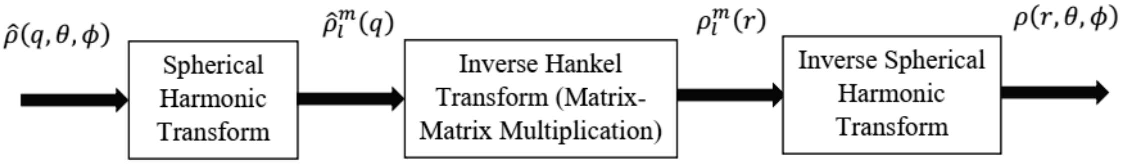

Figure S7 Flowchart of Inverse Polar Fourier Transform.

Table S1 CUDA and HIP syntax similarities

**CUDA API and Library Syntax**	**HIP API and Library Syntax**
`cudaMalloc`	`hipMalloc`
`cudaMemcpy`	`hipMemcpy`
`cudaDeviceSynchronize`	`hipDeviceSynchronize`
`cublasDgemm`	`hipblasDgemm`
`cublasSetStream`	`hipblasSetStream`
`cufftExecZ2Z`	`hipfftExecZ2Z`
`cufftDoubleComplex`	`hipfftDoubleComplex`

S1. Computing *external* FXS data

The angular correlation function can be derived (Supplementary Fig. S1) from N diffraction images I_i, where $i = 1,2,\ldots,N$ as represented below

$$C_{avg}(q, q', \Delta\phi) = \frac{1}{2\pi N}\sum_{i=1}^{N}\int_0^{2\pi} I_i(q,\phi)I_i(q',\phi+\Delta\phi)d\phi, \quad (S1)$$

where I_i is the ith diffraction image, N is the total number of diffraction images, q and q' are a pair of radial coordinates in Fourier space, ϕ is the angle about the incident beam axis, and $\Delta\phi$ is the change in the angle ϕ. The angular correlation function can be related to the intensity spherical harmonics using the Legendre series expansion

$$C_{avg}(q, q', \Delta\phi) = \sum_{l=0}^{\infty} F_l(q, q', \Delta\phi)B_l(q, q'), \quad (S2)$$

$$F_l(q, q', \Delta\phi) = \frac{1}{4\pi}P_l(cos\theta_q cos\theta_{q'} + sin\theta_q sin\theta_{q'} cos\Delta\phi)$$

where, $B_l(q, q')$ is the Legendre expansion coefficients of the angular correlations, P_l is the lth-order Legendre polynomial, $\theta_q = \arccos(q\lambda/(4\pi))$, and λ is the X-ray wavelength. The Legendre coefficients of the angular correlations which represent the *external* FXS data $B_l^e(q, q')$ can be obtained via

$$B_l^e(q, q') = \sum_{\Delta\phi}\{F'\}_{l\phi}(q, q')\, C_{avg}(q, q', \Delta\phi), \quad (S3)$$

where, $\{F'\}_{l\phi}(q, q')$ is the pseudoinverse of the matrix representation of $F_l(q, q', \Delta\phi)$ indexed by degree l and $\Delta\phi$.

S2. Spherical Harmonic Transform

$$f_l^m(r) = \int_0^{2\pi}\int_0^{\pi} f(r,\theta,\phi)\, Y_l^{m*}(\theta,\phi) sin\theta d\theta d\phi, \quad (S4)$$

$$f_l^m(r) = \int_0^{\pi} f_m(r,\theta)P_l^m(cos\theta)sin\theta d\theta, \quad (S5)$$

$$f_m(r,\theta) = \int_0^{2\pi} f(r,\theta,\phi)e^{-im\phi}d\phi, \quad (S6)$$

where, Y_l^m are the spherical harmonics, $f_l^m(r)$ are the spherical harmonic coefficients of the function $f(r,\theta,\phi)$, P_l^m are the associated Legendre functions, l and m are referred as order of the associated Legendre polynomial and $f_m(\theta)$ is an integral on ϕ computed using the Fourier transform.

S3. Inverse Spherical Harmonic Transform

$$f(r,\theta,\phi) = \sum_{l=0}^{\infty}\sum_{m=-l}^{l} f_l^m(r) Y_l^m(\theta,\phi), \quad (S7)$$

$$f(r,\theta,\phi) = \sum_{m=-N}^{N} f_m(r,\theta)\, e^{im\phi}, \quad (S8)$$

$$f_m(r,\theta) = \sum_{l=|m|}^{\infty} f_l^m(r)\, P_l^m(cos\theta), \quad (S9)$$

where, Y_l^m are the spherical harmonics, $f_l^m(r)$ are the spherical harmonic coefficients of the function $f(r,\theta,\phi)$, P_l^m are the associated Legendre functions, l and m are referred as degree and order of the associated Legendre polynomial and $f_m(\theta)$ is an integral on ϕ computed using the Fourier transform.

S4. Correlation Projection

In correlation projection, the *computed* FXS data is scaled to *external* FXS data. The intensity spherical harmonic coefficients are then projected to the closest function consistent with the FXS data such that they satisfy Equation 1. The intensity spherical harmonic coefficients are related to the eigendecomposition of the B_l (represented as a matrix indexed by q and q') for fixed l.

$$B_l = Q_l \Lambda_l Q_l^*, \quad (S10)$$

$$I_l = Q_l \sqrt{\Lambda_l} U_l, \quad (S11)$$

where, B_l is the FXS data decomposed, Q_l is a matrix of eigen vectors, Λ_l is a diagonal matrix of eigen values, U_l is the unitary matrix which solves the unitary Procrustes problem (described below), and I_l represents the intensity spherical harmonic coefficients for each l (with rows indexed by q and column indexed by m).

In the correlation projection, we are solving an unitary Procrustes problem of finding the unitary matrix U_l that minimizes the difference between I_l and $Q_l\sqrt{\Lambda_l}U_l$, in the Frobenius norm. For representation, let us consider $A_l = Q_l\sqrt{\Lambda_l}$, and $B_l = I_l$. The Procrustes problem (Gower *et. al.,* 2004) and the solution can be represented as

$$\min_{U_l} ||A_l U_l - B_l||_F$$

$$X_l = A_l^* B_l$$

$$X_l = P_l \Sigma_l Q_l^*$$

$$U_l = P_l Q_l^*$$

Where, P_l and Q_l are unitary matrices, and Σ_l is the diagonal matrix with non-negative real numbers obtained by the SVD decomposition of X_l.

The matrix in Equation S11 is reassembled to form the modified intensity spherical harmonic coefficients $I_{lmod}^m(q)$ which is used in the inverse direction.

S5. Comments on the number of radial and angular nodes

The number of radial nodes required is related to both the resolution of the reconstruction as well as the oversampling rate of the correlation data. At minimum, phasing requires that the data be oversampled by a factor of two, i.e. the number of radial nodes should be at least twice the number of Shannon channels sampled in the correlation data. However, additional oversampling can improve the robustness of the reconstruction process, especially in the presence of missing data that typically occurs near $q = 0$ due to either a beam stops or gaps in the detector. In the presented reconstructions, the range of radial nodes between N = 80 and 95 oversamples the experimental data by a factor of about four, allowing for a robust and accurate reconstruction in the absence of a very low-q information in the experimental data. Increasing the number of nodes further significantly increases the computational and memory load on CPUs/GPUs without any significant improvement to the quality of the reconstruction.

The main reason 15 is used as a constant for the number of inclination angles in the paper is to be consistent with the PBCV reconstruction results in the PNAS publication. In addition, for numerical experiments of repeatedly applying the polar transform and its inverse, 15 provided near-optimal accuracy and stability. If the constant is too small, the inner spheres of the real-space polar grid have an insufficient number of quadrature points to accurately compute the spherical harmonic transform used in the polar transform. If the constant is too big, the number of computations rise, and could possibly introduce some bias in the reconstruction as the density of grid points would be much greater near the center of the polar grid.
